# Experimental infection of dogs with a feline endogenous retrovirus RD-114

**DOI:** 10.1186/1751-0147-53-3

**Published:** 2011-01-27

**Authors:** Rie Narushima, Noriyuki Horiuchi, Tatsufumi Usui, Takashi Ogawa, Toshio Takahashi, Tomoaki Shimazaki

**Affiliations:** 1 National Veterinary Assay Laboratory, Ministry of Agriculture, Forestry and Fisheries, 1-15-1 Tokura, Kokubunji, Tokyo 185-8511, Japan; 2 Avian Zoonosis Research Center, Faculty of Agriculture, Tottori University, Koyama, Tottori 680-8553, Japan; 3 Department of Veterinary Science, Nippon Veterinary and Life Science University 1-7-1, Kyonan-cho, Musashino-shi, Tokyo 180-8602, Japan; 4 Animal Health Division, Food Safety and Consumer Affairs Bureau, Ministry of Agriculture, Forestry and Fisheries, 1-2-1 Kasumigaseki, Chiyoda-ku, Tokyo 100-8950, Japan

## Abstract

**Background:**

The feline endogenous retrovirus RD114 is contained in the genome of cats. The virus may contaminate live canine vaccines based on cultured feline cells. The *in vivo *infectivity, acute and subacute pathogenicity, and viral proliferation of the RD114 virus were evaluated by experimental infection of dogs.

**Methods:**

Nine specific pathogen free dogs were divided into three groups, with each group consisting of one female and two male dogs. Dogs were subcutaneously inoculated in the neck with either 1 ml RD114 stock virus (group A), inactivated RD114 virus suspension (group B), or cell culture medium (group C) as a negative control. To assess blood cell counts and biochemical properties, blood samples from each group were collected 5 days before inoculation, just prior to inoculation, and 1, 3, 7 and 10 days post-inoculation.

**Result:**

During the experimental period of 51 days, none of the dogs inoculated with RD114 virus showed any clinical signs, significant increases in rectal temperature or abnormal blood biochemical characteristics including C-reactive protein when compared with the negative controls. We were not able to re-isolate the RD114 virus from buffy coat cells of group A dogs. Additionally, we could not detect RD114 provirus in the genomic DNA isolated from peripheral blood leukocytes, lymph node, spleen and sternal bone marrow cells.

**Conclusions:**

Signs of RD114 virus proliferation were not found after subcutaneous infection of dogs. Although the potential risk caused by infection with RD114 virus in dogs could not be assessed in this study, we suspect that RD114 virus has little or no virulence in dogs.

## Background

Domestic cats are generally assumed to harbour the infectious endogenous retrovirus RD114 in their genome [1,2]. It is known that the Crandell-Rees feline kidney cell line is contaminated with an RD114-like virus [3]. Recently, Miyazawa *et al*. [4] found that certain live attenuated vaccines for dogs were contaminated with infectious RD114 virus. We also confirmed in our laboratory that infectious RD114 virus was present in certain live attenuated canine vaccines that were manufactured using feline cells (unpublished data). The amount of infectious RD114 virus found in manufactured live canine vaccines was as high as 1,800 50% tissue culture infective dose (TCID_50_)/vial (one vial represents a single dose) [4]. RD114 virus can be regarded as an 'exogenous' retrovirus in non-feline species including dogs, however there is no information concerning the etiological features of RD114 virus infection in dogs. The present study was conducted to evaluate the *in vivo *infectivity, acute and subacute pathogenicity, and viral proliferation of the RD114 virus by experimental infection of specific pathogen free (SPF) dogs.

## Methods

### Virus preparation

A LacZ marker rescue assay was used to detect and titrate infectious RD114 virus [5,6]. The principle of the assay is based on the detection of infectious RD114 virus using TE671 (human rhabdomyosarcoma) cells transduced with the LacZ marker gene [TE671(LacZ) cells]. RD114 virus was prepared from the culture supernatant of TE671 cells chronically infected with the virus [7]. Culture supernatants were filtered through a 0.45 μm pore size membrane filters, and aliquots stored at -80 °C until required. The titre of the stock virus was approximately 10^5 ^infectious units/ml. To prepare inactivated RD114 virus as inocula, the stock virus was added to an equal volume of diethylether, then vigorously and intermittently mixed at room temperature (around 20°C) for 3 min. Absence of infectious RD114 virus in the inactivated viral preparation was confirmed by the LacZ marker rescue assay.

### Dog inoculation

Nine 10-months-old SPF beagles (six males and three females) were divided into three groups (groups A, B and C). Each group consisted of one female and two male dogs with individual dogs confined to cages. The dogs were inoculated subcutaneously in the neck with either 1 ml of RD114 stock virus (group A), inactivated RD114 virus suspension (group B) or cell culture medium (group C) as a negative control. The inoculation route was chosen according to the manufacturer's instructions for the live canine vaccines. All animal studies were conducted in accordance with the National Veterinary Assay Laboratory Guide for the Care and Use of Laboratory Animals, and the relevant Animal Welfare Acts. The dogs were euthanized 51 days post inoculation (PI) and autopsied. The thoracic organs, abdominal organs and bone marrow were macroscopically examined. The axillary lymph nodes, spleen and sternal bone marrow were collected for further virological examinations.

### Blood analyses and clinical examination

Re-isolation of the RD114 virus was attempted by co-culturing TE671(LacZ) cells with buffy coat cells. To assess the blood cell counts and biochemical properties, blood samples from each group were collected 5 days before inoculation, just prior to inoculation (day 0), and at 1, 3, 7 and 10 days PI. White blood cells (WBC), red blood cells, haemoglobin, haematocrit, mean cell volume, mean corpuscular haemoglobin, mean corpuscular haemoglobin concentration, and platelet numbers were determined in the blood cell counts. The biochemical analyses included total protein, albumin, total bilirubin, glutamic-oxaloacetic transaminase, glutamic-pyruvic transaminase, alkaline phosphatase, lactic dehydrogenase, amylase, lipase, blood urea nitrogen, creatinine, total cholesterol, triglyceride, sodium, potassium, chlorine, calcium, inorganic phosphorous, glucose and total bile acid levels. The C-reactive protein (CRP) concentration, a sensitive indicator of inflammation in dogs, was analysed at days 0, 7, and 51 PI. Additionally, during the experimental period, clinical signs were recorded daily, and rectal temperature and body weight were measured at least once a week.

### Polymerase chain reaction

Genomic DNA was extracted from ethylenediaminetetraacetic acid (EDTA) stabilized whole blood, axillary lymph nodes, spleen and bone marrow, and subjected to polymerase chain reaction (PCR) analysis. In addition, genomic DNA was also extracted from TE671(LacZ) cells, 12 days after co-culture with buffy coat cells. The PCR was conducted as described by Sakaguchi *et al*. [6] by amplifying a portion of the *env *and *pol *genes of RD114 virus. The single step PCR assay was performed on genomic DNA extracted from TE671(LacZ) cells infected with ten-fold serially diluted (10^0 ^to 10^-10^) stock virus. The limit of detection was 10^-5 ^and 10^-6 ^for the *env *and *pol *genes, respectively. Then, the LacZ pseudotype virus positive cells were detected 17, 2 and 0 cells for 10^-4^, 10^-5 ^and 10^-6 ^diluted points, respectively.

### Statistical analysis

The mean values of all measured variables of each group were calculated. Statistical analyses were performed using SPSS software (version 13). The differences between the three groups at each time point were compared with *P *values of <0.05 considered to be significant.

## Results

**Figure 1:**
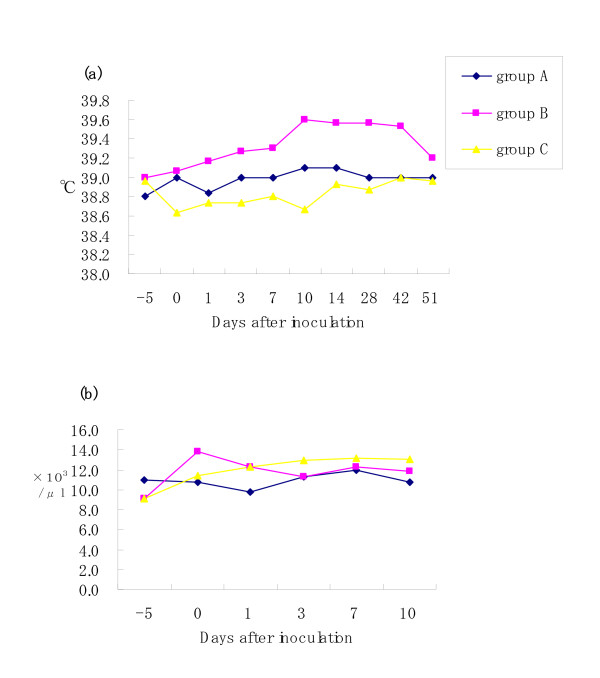
**Mean rectal temperatures (a) and white blood cell counts (b)**. Significant changes in rectal temperature or white blood cell counts in 3 groups of dogs (n = 3 for each group). Group A, RD114 virus inoculated group; B, inactivated virus group; C, control group.

During the experimental period, none of the dogs in groups A and B showed any clinical signs, including significant increases in rectal temperature, or distinct abnormal biochemical blood characteristics including CRP when compared with group C dogs. In addition, the WBC counts of group A and B dogs were nearly equal to those of group C, and body weight increased in all animals after inoculation (Figure 1). Statistical analysis showed that the only significant difference was seen in the potassium level in the three groups five days before inoculation. At autopsy (51 days PI), any significant lesions were not observed macroscopically. Major lymph nodes, especially the axillary lymph nodes, were small, ranging from 0.5-1 cm in diameter with distinct cortico-medullary junctions. The spleen and bone marrow did not contain any significant lesions.

**Figure 2:**
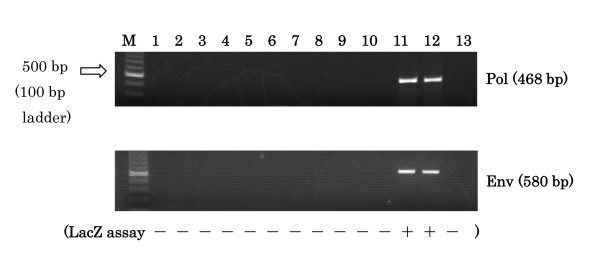
**Polymerase chain reaction (PCR) analysis of RD114 virus in buffy coats**. Genomic DNA extracted from the buffy coat samples of dogs (n = 3 for each group) in three groups (A: RD114 virus inoculated group; B: inactivated virus group; C: control group) was tested for the presence of RD114 virus. Lanes 1-3, group A; 4-6, group B; 7-9, group C; 10, negative control (medium); 11, positive control 1 (RD114 stock virus); 12, positive control 2 (buffy coat of group C mixed with RD114 stock virus); 13, negative control (distilled water). No PCR positives were obtained from any of the experimental groups A-C.

Infectious RD114 virus was not detected from TE671(LacZ) cells co-cultured with buffy coat cells using the LacZ marker rescue assay. This finding was also confirmed by PCR assays using genomic DNA from the co-cultured cells as a template. Additionally, RD114 provirus was not detected in genomic DNA extracted from peripheral blood, lymph nodes, spleen and sternal bone marrow, using the one-step PCR assays (Figure. 2).

## Discussion

It has been reported that RD114 virus infects a variety of canine cell lines, such as the Mardin-Darby canine kidney line [8]. RD114 virus also actively infects cells from cats and dogs, and may be transmitted to non-feline species because of the xenotropic features of the virus *in vitro *[9]. Therefore, if dogs are exposed to a high dose of RD114 virus, the viral genome may integrate into the cells of target tissues. Viraemia is often associated with the acute phase of viral diseases. In clinical cases caused by retroviruses, blood specimens are mainly used for virus detection. However, we were unable to detect RD114 provirus from blood, lymph node, spleen and sternal bone marrow samples using a one-step PCR assay, despite group A dogs being inoculated with approximately 10^5 ^infectious units of RD114 virus. These results suggest that RD114 did not proliferate and disseminate in the dogs, or that RD114 virus did not proliferate efficiently in the blood cells and hematopoietic system of dogs. The virus stock, neutralized with the sera of each dog, was inoculated onto TE671(LacZ) cells, and the number of lacZ positive foci among the three groups were counted. In the present study, the antibody titres remained unchanged in all dogs following inoculation (data not shown), implying that RD114 virus did not proliferate in dogs.

The findings indicate that the RD114 virus has little or no virulence in dogs. The potential risk caused by infection with RD114 virus in dogs cannot be accurately assessed because a longer PI period is required for the verification or exclusion of retroviral infection. It is also possible that dogs younger than 10-months might be more susceptible to RD114 virus infection. Millions of puppies are vaccinated worldwide on an annual basis. Therefore it is impossible to completely rule out adverse effects of the infection. Many exogenous retroviruses cause leukaemia and tumours in a wide variety of animal species, and further research is required to isolate or detect RD114 virus in dogs that have developed leukaemia and tumours following use of live canine vaccines manufactured using feline cells. Hopefully, such research would clarify the possible relationship between the occurrence of diseases in dogs and vaccination.

## Conclusions

Signs of RD114 virus proliferation were not found after subcutaneous infection of dogs. Although the potential risk caused by infection with RD114 virus in dogs could not be assessed in this study because the minimum infectious dose and pathogenicity remain unknown, we suspect that RD114 virus has little or no virulence in dogs.

## Abbreviations

SPF: Specific pathogen free. The dogs were free from infections with canine adenovirus (CAdV-2), canine coronavirus, canine distempervirus, canine parainfluenzavirus, canine parvovirus, leptospira and rabies virus.

## Competing interests

The authors declare that they have no competing interests.

## Authors' contributions

RN participated in the design of the study and performed the statistical analysis. NH performed autopsy. TU organised basic equipment. TO participated in collecting blood samples. TT performed finalisation of the manuscript. TS conceived of the study, and participated in its design and coordination. All authors read and approved the final manuscript.
